# Effects of ASC Application on Endplate Regeneration Upon Glycerol-Induced Muscle Damage

**DOI:** 10.3389/fnmol.2020.00107

**Published:** 2020-06-23

**Authors:** Matteo Rigon, Sarah Janice Hörner, Tatjana Straka, Karen Bieback, Norbert Gretz, Mathias Hafner, Rüdiger Rudolf

**Affiliations:** ^1^Institute of Molecular and Cell Biology, Mannheim University of Applied Sciences, Mannheim, Germany; ^2^Institute of Transfusion Medicine and Immunology, Medical Faculty Mannheim, Heidelberg University, Mannheim, Germany; ^3^Medical Research Center, Medical Faculty Mannheim, Heidelberg University, Mannheim, Germany; ^4^Institute of Medical Technology, Medical Faculty Mannheim, Mannheim University of Applied Sciences, Mannheim, Germany; ^5^Interdisciplinary Center for Neurosciences, Heidelberg University, Heidelberg, Germany

**Keywords:** glycerol, skeletal muscle, NMJ, ASC, regeneration

## Abstract

Amongst other approaches, adipose-derived stromal cells (ASCs) have recently been tested with respect to their regenerative capacity for treatment of neuromuscular disorders. While beneficial effects of ASCs on muscle recovery were observed previously, their impact on regeneration of neuromuscular junctions (NMJs) is unclear. Here, we used a murine glycerol damage model to study disruption and regeneration of NMJs and to evaluate the effects of systemic application of ASCs on muscle and NMJ recovery. In mice that were not treated with ASCs, a differential response of NMJ pre- and post-synapses to glycerol-induced damage was observed. While post-synapses were still present in regions that were necrotic and lacking actin and dystrophin, pre-synapses disappeared soon in those affected areas. Partial regeneration of NMJs occurred within 11 days after damage. ASC treatment slightly enhanced NMJ recovery and reduced the loss of presynaptic sites, but also led to a late phase of muscle necrosis and fibrosis. In summary, the results suggest a differential sensitivity of NMJ pre- and post-synapses to glycerol-induced muscle damage and that the use of ASC for the treatment of neuromuscular disorders needs further careful evaluation.

## Introduction

Despite decades of research and numerous trials, the treatment options for most neuromuscular disorders are still disappointing. While the number of drugs approved or in clinical trials has strongly risen in the past decade (Cowling and Thielemans, 2020) the development of stem-cell based therapies is another important line of activity in this context. The hope is, that stem cells might enable direct tissue regeneration *in vivo* (Gois et al., 2019). At present, different stem cell sources are being tested, including autologous or heterologous muscle-derived stem cells, mesangioblasts, muscle-derived progenitors, interstitial cells, and mesenchymal stromal/stem cells (Gois et al., 2019). The latter group includes adipose-derived stromal cells (ASCs), that are interesting as a source of stem cells, because besides being immune-privileged they are readily accessible from lipoaspirates and can be easily isolated and expanded in vitro (Kern et al., 2006; Torres-Torrillas et al., 2019). It remains open whether their local or systemic application might be more beneficial for the treatment of neuromuscular disorders. While local injection might come with a higher potential for targeted cell integration, it is difficult to achieve for diseases where different organs or large parts of the body are affected, as it is true for most neuromuscular disorders (Gois et al., 2019). On the other hand, while systemic application might have a drawback in the entrapment of most injected cells in organs such as lungs and liver (Gois et al., 2019) recent studies suggest that a principal function of stem cell therapies relies in their modulatory activities on cellular signaling rather than direct cell engraftment (Gorecka et al., 2018; Boldyreva et al., 2019; Mitchell et al., 2019; Torres-Torrillas et al., 2019). Although in different animal models and clinical applications beneficial effects of stem cell grafts on recovery of muscle tissue were reported (Forcales, 2015; Torres-Torrillas et al., 2019), little emphasis has been put on their impact at the level of the neuromuscular junction (NMJ).

In order to study skeletal muscle recovery mechanisms, several muscle damage models have been developed (Arnold et al., 2007; McCarthy et al., 2011; Czerwinska et al., 2012; Iwata et al., 2013; Tiryakioglu et al., 2015; Hardy et al., 2016; Mahdy, 2018). Those models differ in the injury mechanism and in the type of harm caused, affecting the degrees of degeneration and regeneration capabilities of the muscle. Among those, glycerol was reported to cause a damage by osmotic shock, affecting not only myofibers but also other muscle elements, including the basal lamina (Kawai et al., 1990; Pisani et al., 2010; Mahdy et al., 2015). This leads to an impaired regeneration with ectopic infiltration of adipocytes and fibrosis (Lluís et al., 2001; Mahdy et al., 2015, 2016; Hardy et al., 2016) and deprives the regenerating muscle cells of the interaction with extra-cellular matrix (ECM) elements that can enhance tissue recovery (Murphy et al., 2011; Iwata et al., 2013; Webster et al., 2015). Although characterized by a severe initial destruction, muscles treated with intra-muscular glycerol injection are able to recover anyway (Arsic et al., 2004; Mahdy et al., 2015, 2016). This suggested that glycerol injection might be a model well suited for the study of muscle regeneration. Accordingly, to investigate de- and regeneration of NMJs in the absence and presence of systemic ASC administration, a glycerol-induced muscle damage model was chosen. Upon toxin- or chemical-induced injury, muscle necrosis and fibrosis take place. Necrotic muscle cells, due to their compromised plasma membrane, allow the uptake of serum proteins, including immunoglobulins, into the cytosol (Iwata et al., 2013; Judson et al., 2013; Proto et al., 2015; Arecco et al., 2016; Rodrigues et al., 2016), rendering immunofluorescence against immunoglobulin G (mIgG) useful to identify necrotic areas. Necrotic leakage and linked degradative processes can also lead to the loss of dominant cytosolic skeletal muscle proteins, such as f-actin (Huang and Gopalakrishnakone, 1996) and dystrophin (Biral et al., 2000). Muscle fibrosis is characterized by deposition of collagen type I (Gibertini et al., 2014), one of the major components of ECM (Huebner et al., 2008; Serrano and Muñoz-Cánoves, 2010; Gillies and Lieber, 2011; Feng et al., 2019). Following muscle degeneration, muscle recovery involves detection of regenerating myofibers, which are characterized by the expression of embryonic myosin heavy chain (eMHC, also known as myosin 3) (Schiaffino et al., 1986; Murphy et al., 2011; Judson et al., 2013; McDonald et al., 2015; Kastenschmidt et al., 2019) and by nuclei in central position (in the following, referred to as center-nucleated fibers, CNFs) (Judson et al., 2013; McDonald et al., 2015; Arecco et al., 2016; Kastenschmidt et al., 2019). In order to monitor NMJ degradation and recovery, markers for pre- and post-synapses were analyzed. While vesicular acetylcholine transporter (VAChT) is localized to small synaptic vesicles at the endplate terminal (Maeda et al., 2004; Sugita et al., 2016), acetylcholine receptor (AChR) is highly enriched at the postsynaptic membrane of NMJs (Fambrough et al., 1979).

The present study aimed at developing and characterizing a reliable glycerol-based de/regeneration model and to apply this for the analysis of potential effects of ASCs on regeneration of muscle and NMJs. Using the glycerol damage paradigm, we obtained differential profiles for pre- and postsynaptic destruction and recovery. While post-synapses appeared to remain relatively stable even upon heavy muscle fiber damage and loss of internal structures, presynaptic contacts were severely affected upon glycerol injection and needed to recover over a couple of days. Treatment with ASCs led to a slightly reduced loss of post-synapses and improvement of pre- to postsynapse match upon glycerol treatment, but these intermediate effects were reverted during a second phase of inflammation.

## Materials and Methods

### Glycerol and Stem Cell Injection

All animal experiments were conducted in accordance with the German Animal Protection Law and approved by the local authority (Regierungspräsidium Nordbaden, Karlsruhe/Germany in agreement with EU guideline 2010/63/EU; license G-139/18). In order to induce muscle damage, 20 μl of a 50% glycerol solution diluted in physiological saline were injected into the tibialis anterior (TA) muscle of anesthetized 2 months old C57BL6 female mice. The decision to use female arose from two observations: first, females are experimentally easier to handle, since animals should preferentially be kept in groups for social interactions, but males under these circumstances often bite each other and this can lead to damage of muscles that is not due to the experimental protocol. Second, females seem to possess a slightly better regenerative capability (Deasy et al., 2007; Kitajima and Ono, 2016). The same volume of saline solution was injected contralaterally as a sham. The animals were then euthanized by cervical dislocation after 18 h or 3, 5, 8, or 11 days following the procedure and the muscles were dissected. Where ASCs were applied, one million cells diluted in 100 μl of sterile PBS where injected via tail vein directly after glycerol treatment. In this case, animals were euthanized by cervical dislocation and dissected 3, 5, and 11 days after the injection. At least three animals for each timepoint and condition (glycerol and ASC with glycerol) were analyzed.

### Human Adipose-Derived Mesenchymal Stem Cells

Adipose-derived stromal cells were isolated from adult adipose tissue obtained from lipoaspirates of healthy donors, after obtained informed consent (Mannheim Ethics Commission II vote numbers 2010-262 N-MA, 2009-210 N-MA, 49/05 and 48/05), as described before (Bieback et al., 2012). Briefly, the lipoaspirate was washed with sterile PBS to remove cellular debris and red blood cells and digested with 0.15% w/v collagenase type I (Sigma-Aldrich, Munich, Germany), for 45 min at 37°C. After washing, the pellet was resuspended in medium (DMEM low glucose, 10% human AB serum, 100 U/ml penicillin, 0.1 mg/ml streptomycin and 4 mM L-glutamine), plated and incubated overnight at 37°C, 5% CO_2_. After 1 day, the non-adherent and red blood cells were removed. Expanded cells were characterized regarding their proliferation capacity, immune phenotype, and adipo- and osteogenic differentiation potential, as described previously (Bieback et al., 2012). Before injection, ASCs were seeded at 750 ASC/cm^2^ and expanded. On injection days, ASCs were trypsinized for 5 min at 37°C and washed once with medium. The ASC pellet was then resuspended in sterile PBS at a concentration of 1 × 10^7^ cells/ml shortly before the injection.

### Immunofluorescence Analysis

Freshly after dissection, TA muscles were covered with a thin layer of Frozen Section Compound (Leica) and frozen on liquid nitrogen. Transverse cryosections of 15–20 μm thickness were cut using a Leica CM1950 cryostat, carefully positioned on glass microscope slides (Thermo Fisher Scientific) and then immunostained for epitope detection. Cryosections were permeabilized with 0.1% TritonX-100/PBS for 10 min at room temperature and, after three washing steps in PBS (5 min each), incubated with 2% BSA/PBS for 2 h to avoid unspecific binding of primary antibodies. The sections were then incubated overnight at +4°C with primary antibodies diluted in 2% BSA/PBS. After three washing steps in 2% BSA/PBS, sections were incubated with the secondary antibodies/dyes/toxins in 2% BSA/PBS for 3 h at room temperature and in the darkness. Antibodies, dyes, and dilution rates are listed in Table 1. Subsequently, after 15 min incubation with DAPI (Sigma) in 2 % BSA/PBS and three more washing steps with PBS, glass slides were left to briefly dry and then mounted in Mowiol. The samples were then analyzed at the confocal microscope the day after. Images were taken using a Leica TCS SP8 microscope equipped with 405, 488, 555, and 633 nm lasers, and Leica HC PLAN APO 20×/0.75 IMM CORR CS2 objective and at z-steps of 3 μm. For every sample, three to five entire cryosections from the central portion of the muscle at an intersection interval of roughly 200 μm were visualized. Staining of ASCs used a slightly modified protocol as follows. Cells on Eppendorf 8 chamber cell imaging slides were briefly fixed with 2% PFA/PBS and then permeabilized with 0.1% TritonX-100/PBS for 5 min at room temperature and, after three washing steps in PBS (5 min each), incubated with 2% BSA/PBS for 1 h. Subsequently, cells were incubated overnight at 4°C with primary antibody diluted in 2% BSA/PBS. After three washing steps in 2% BSA/PBS, cells were incubated with the secondary antibody and DAPI diluted in 2% BSA/PBS for 45 min and in the darkness. After three more washing steps with PBS and one with ddH_2_O, the slides were mounted in Mowiol and analyzed using the same microscope and objective used for the muscle cryosections. Antibodies, dyes, and dilution rates are listed in Table 1.

**TABLE 1:** List of used antibodies and dyes.

	Company	Catalog number	Lot number	Dilution rate
**Primary antibody**				
Anti-Dystrophin antibody	Invitrogen	PA 1-21011	TK2665551J	1:200
Anti-Myosin 3 antibody	Biorbyt	orb385438	RBQ34	1:100
Anti-VAChT antibody	Synaptic Systems	139 103	2-44	1:500
Anti-Collagen type I antibody	Rockland	600-401-103-0.1	40681	1:500
Recombinant anti-Ku80 antibody (EPR3468)	Abcam	GR3216586-3	ab80592	1:300
**Secondary antibody/marked toxins/dyes**				
Donkey anti-Rabbit IgG (H + L) Highly Cross-Adsorbed Secondary Antibody, Alexa Fluor 488	Thermo Fisher Scientific	A-21206	176375	1:500
Donkey anti-Rabbit IgG (H + L) Highly Cross-Adsorbed Secondary Antibody, Alexa Fluor 488	Thermo Fisher Scientific	A-21206	176375	1:500
Goat anti-Rabbit IgG (H + L) Cross-Adsorbed ReadyProbes Secondary Antibody, Alexa Fluor 594	Thermo Fisher Scientific	R37117	1875978	1:500
F(ab’)2-Goat anti-Rabbit IgG (H+L) Cross-Adsorbed Secondary Antibody, Alexa Fluor 647	Thermo Fisher Scientific	A-21246	55002A	1:500
Goat anti-Mouse IgG (H + L) Highly Cross-Adsorbed Secondary Antibody, Alexa Fluor 555	Thermo Fisher Scientific	A-21424	1802436	1:500
Goat anti-Mouse IgG (H + L) Cross-Adsorbed Secondary Antibody, Alexa Fluor 488	Thermo Fisher Scientific	A-11001	1834337	1:500
α-Bungarotoxin, Alexa Fluor 488 conjugate	Thermo Fisher Scientific	B13422	1750294	1:500
α-Bungarotoxin, Alexa Fluor 555 conjugate	Thermo Fisher Scientific	B35451	1880574	1:500
Alexa Fluor 555 Phalloidin	Thermo Fisher Scientific	A34055	1780358	1:500
DAPI	Sigma-Aldrich	D9542	28114320	1:1000

### Semi-Automated Quantification

Confocal images obtained from muscles cryosections were processed and analyzed using ImageJ (FIJI version). From maximum projections of the images, areas which showed the presence of mIgG, collagen I, or actin were manually thresholded, areas selected and measured. The entire area of the cryosection was contoured by hand using the background signal. The areas where dystrophin-outlined cells were present were obtained from maximum projected confocal images to which a median filter was applied and then thresholded. Subsequently the “analyze particles” function of ImageJ was used (selecting 20 μm as lowest value and including holes). The image obtained was then measured. Muscle fibers, CNFs, and eMHC+ fibers were counted manually. For each sample, a minimum of three cryosections was processed and analyzed. NMJs, identified by AChR and VAChT expression together with their shape and/or position with respect to muscle fibers, were analyzed and quantified manually. Where indicated, the areas positive for AChR and/or VAChT (per endplate) were thresholded, segmented and measured. Numbers of analyzed synapses are given in Supplementary Table S1. Signals for pre- and post-synapses obtained from three different cryosections per sample were analyzed. For the Ku80 detection, DAPI signals corresponding to cell nuclei were thresholded and segmented. A selection created from those signals was then used to hand-segment the Ku80+ signals, selecting those areas where it was colocalizing within cell nuclei.

### Statistical Analysis and Figure Compilation

For statistical analysis, data were first screened for normality and homoscedasticity using Kolmogorov–Smirnov test and F-test. Mean and SD/SEM were calculated. Then, significance was assessed with Student’s *t*-test or Welch test. Data compilation was done in Microsoft Excel for Mac, plots were made using either Microsoft Excel for Mac or Graph Pad Prism. Figures were prepared in Adobe Photoshop and then compiled in Adobe Illustrator.

## Results

### Glycerol Injection Leads to Robust Muscle Necrosis With Fast Regeneration

**FIGURE 1:**
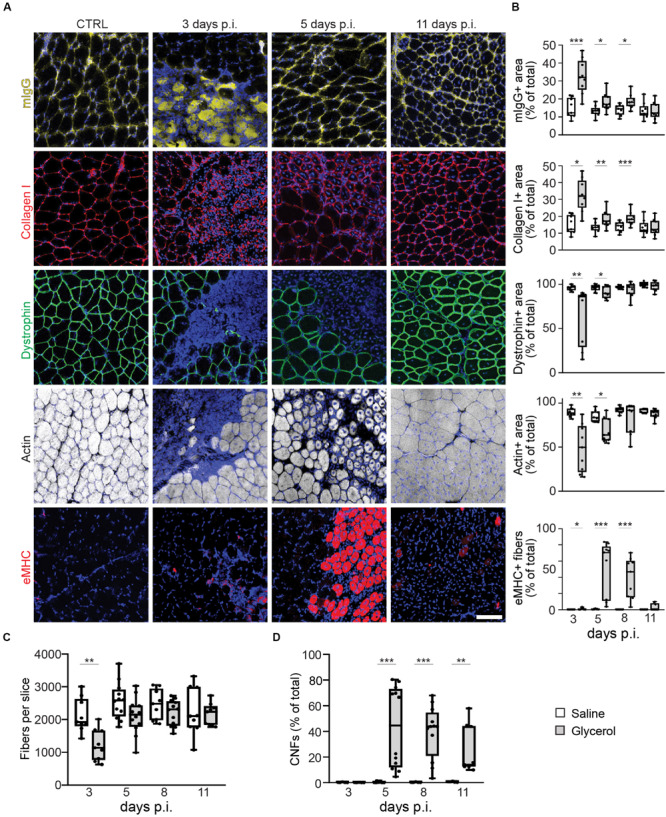
Glycerol-induced muscle damage leads to early necrosis, loss of dystrophin and actin, transient eMHC appearance, and prolonged presence of center-nucleated fibers. TA muscles were injected with 20 μl of either saline or glycerol and then harvested and snap frozen after3, 5, 8, or 11 days post-injection (days p.i.). Upon cryosectioning, muscle slices were stained with DAPI and either antibodies against mouse IgG (mIgG), collagen I, dystrophin, or embryonic myosin heavy chain (eMHC), or with phalloidin-TRITC to label actin. Sections were analyzed by confocal microscopy. **(A)** Representative optical sections of fluorescence signals as indicated, nuclear DAPI staining always shown in blue, mIgG in yellow, collagen I and eMHC in red, dystrophin in green, actin in gray. CTRL, saline-injected muscles at 3 days p.i., the other panels depict glycerol-injected muscles at3, 5, and 11 days p.i., as indicated. Scalebar, 100 μm. **(B)** Quantitative analysis of section areas positive for fluorescence signals of either mIgG, collagen I, dystrophin, or actin, or number of eMHC-positive fibers, as a function of days p.i. Box–Whisker plots show all individual data points as dots, the extensions of upper and lower quartiles in the boxes, the medians as horizontal lines in the boxes, and maxima and minima as whiskers. *p ≤ 0.05, **p ≤ 0.01, ***p ≤ 0.001. **(C,D)** Quantitative analysis of fibers per muscle slice **(C)** or CNFs (% of fiber number, **D**) as a function of days p.i. Box–Whisker plots show all individual data points as dots, the extensions of upper and lower quartiles in the boxes, the medians as horizontal lines in the boxes, and maxima and minima as whiskers. **p ≤ 0.01, ***p ≤ 0.001.

To understand the effects of glycerol injection on muscle integrity and regeneration, a panel of markers for muscle necrosis, function, and regeneration was analyzed. Therefore, TA muscles were injected with 20 μl of either saline (control) or glycerol and then muscles were harvested after 18 h and 3, 5, 8, and 11 days. Sliced muscles were co-stained with DAPI for nuclei and antibodies against mouse IgG, collagen I, dystrophin, or eMHC to assess the status of necrosis, fibrosis, muscle fiber integrity, and regeneration. Co-staining with phalloidin served to visualize the presence of f-actin. Confocal images of these sections revealed a massive muscle damage already after 18 h of glycerol injection (Supplementary Figure S1). As shown in Figure 1 and Supplementary Figure S2, after 3 days of injury due to glycerol injection involved large parts of muscles and was characterized by passage of immunoglobulins through sarcolemma, enhanced accumulation of collagen I, and loss of the functional markers, dystrophin and f-actin. Subsequently, these signs of muscle damage progressively decreased and were followed by a wave of eMHC expression, principally between days 5 and 8 after glycerol injection. On day 11 after glycerol treatment, muscles had largely reassumed their normal appearance lacking degeneration and regeneration markers and showing regular distribution of dystrophin and f-actin. Also, numbers of muscle fibers, that were significantly decreased on day 3 post glycerol injection, were comparable to saline-treated muscles at day 11 (Figure 1C). Cross-sectional areas of remaining fibers did not vary between the different experimental groups (Supplementary Table S2). The only clear sign of ongoing muscle regeneration at 11 days post injection was the presence of CNFs that rose from less than 1% in control conditions to around 40% on days 5 and 8 after glycerol injection and decreased to roughly 25% on day 11. In summary, the glycerol injection paradigm displayed a simple, regular, and controlled sequence of muscle damage and regeneration. In the following, we used this model to address NMJ degeneration and regeneration.

### Glycerol Injection Induces a Transient Loss of Pre-synapses

**FIGURE 2:**
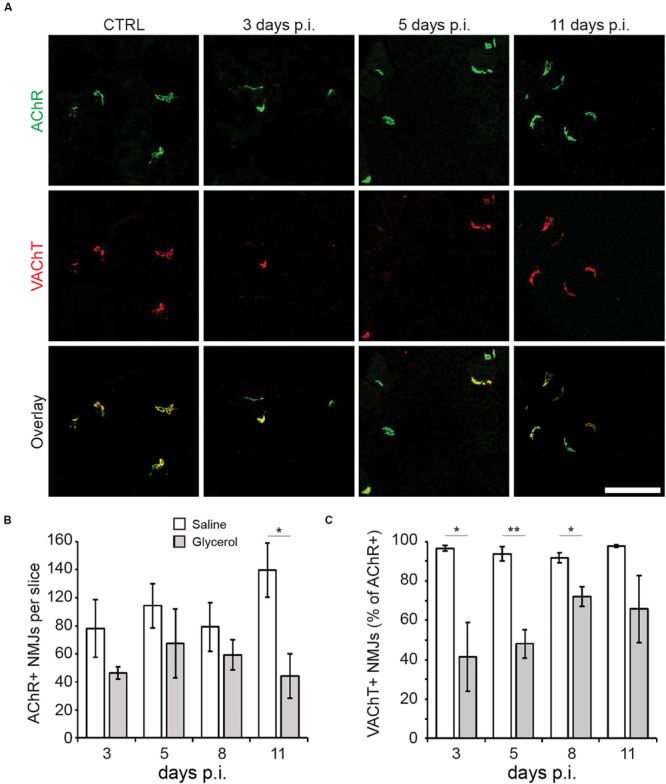
Glycerol injection primarily affects pre-synapses. TA muscles were injected with 20 μl of either saline or glycerol and then harvested and snap frozen after 3, 5, 8, or 11 days (days p.i.). Upon cryosectioning, muscle slices were stained with αBGT (for AChR detection) and antibodies against VAChT to label post- and presynaptic portions of NMJs, respectively. Sections were analyzed by confocal microscopy. **(A)** Representative optical sections of fluorescence signals as indicated, αBGT in green, VAChT in red, yellow in overlay images indicates colocalization of both signals. CTRL, saline-injected muscles at 3 days p.i., the other panels depict glycerol-injected muscles at 3, 5, and 11 days p.i., as indicated. Scalebar, 100 μm. **(B,C)** Quantitative analysis of αBGT+ postsynaptic sites per slice **(B)** and VAChT+ NMJs (% of αBGT+ structures, **C**) as a function of days p.i. Shown is mean ± SEM (*n* = 3 muscles). *p ≤ 0.05, **p ≤ 0.01.

To visualize the effect of glycerol injection on NMJs, TA muscles injected with either glycerol or saline (control) were taken at days 3, 5, 8, and 11 after injection. Slices were stained against postsynaptic nicotinic AChR and presynaptic VAChT with fluorescent α-bungarotoxin (αBGT) and anti-VAChT antibody, respectively. Confocal microscopy analysis of these samples revealed that while AChR+ sites were present at all timepoints, many of these were negative for VAChT at 3, 5, and 8 days post glycerol injection (Figure 2A). Quantitative analysis confirmed that in glycerol-treated muscles, the number of AChR+ sites per sample was slightly but not significantly reduced (Figure 2B) compared to saline-treated controls. The size of synaptic regions was unaltered (Supplementary Table S3). The only exception was at 11 days post injection, where a higher amount of post-synapses was detected in the control samples than at the other timepoints. With respect to VAChT- and AChR-double positive synapses, glycerol-treated muscles showed significantly lower values between days 3 and 8 post injection as compared to saline samples (Figure 2C). These data suggest that glycerol, although affecting both pre- and postsynaptic sites, had a more detrimental impact on the presynaptic component.

### Kinetics of Glycerol-Induced Muscle Damage and Regeneration Are Altered by ASCs

**FIGURE 3:**
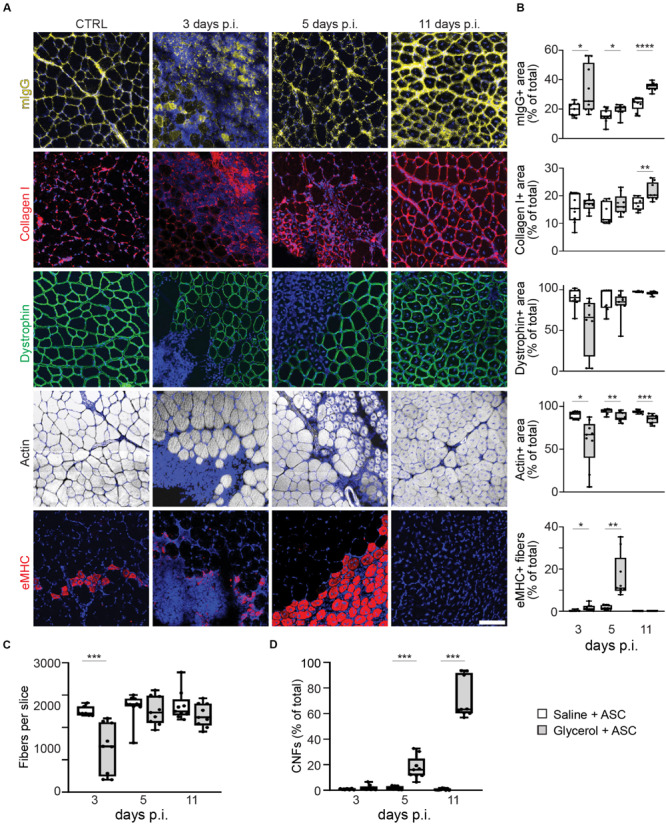
Systemic injection of ASCs induces a late phase of IgG infiltration, enhanced basal eMHC expression, and increase in center-nucleated fibers. Simultaneous to tail-vein injection of ASCs, TA muscles were injected with 20 μl of either saline or glycerol and then harvested and snap frozen after 3, 5, or 11 days (days p.i.). Upon cryosectioning, muscle slices were stained with DAPI and either antibodies against mouse IgG (mIgG), collagen I, dystrophin, or embryonic myosin heavy chain (eMHC), or with phalloidin-TRITC to label actin. Sections were analyzed by confocal microscopy. **(A)** Representative optical sections of fluorescence signals as indicated, nuclear DAPI staining always shown in blue, mIgG in yellow, collagen I and eMHC in red, dystrophin in green, actin in gray. CTRL, saline-injected muscles at 3 days p.i., the other panels depict glycerol-injected muscles at 3, 5, and 11 days p.i., as indicated. Scalebar, 100 μm. **(B)** Quantitative analysis of section areas positive for fluorescence signals of either mIgG, collagen I, dystrophin, or actin, or number of eMHC-positive fibers, as a function of days p.i. Box–Whisker plots show all individual data points as dots, the extensions of upper and lower quartiles in the boxes, the medians as horizontal lines in the boxes, and maxima and minima as whiskers. *p ≤ 0.05, **p ≤ 0.01, ***p ≤ 0.001, ****p ≤ 0.0001. **(C,D)** Quantitative analysis of fibers per muscle slice **(C)** or center-nucleated fibers (% of fiber number) as a function of days p.i. Box–Whisker plots show all individual data points as dots, the extensions of upper and lower quartiles in the boxes, the medians as horizontal lines in the boxes, and maxima and minima as whiskers. ***p ≤ 0.001.

The second goal of this study was to address the effect of ASCs on muscle and NMJ regeneration. Thus, directly after intra-muscular application of either saline or glycerol, ASCs were administered systemically via the tail vein. Then, microscopic analysis was performed to evaluate the consequences of ASC treatment on muscle de- and regeneration. Therefore, muscles were harvested at days 3, 5, and 11 post injection and then sliced. Sections were stained for nuclei and mouse IgG, collagen I, dystrophin, eMHC, or f-actin. While fiber numbers, dystrophin and f-actin results were similar to the samples not treated with ASCs, the data for mIgG, collagen I, eMHC, and CNFs showed interesting differences (Figure 3 and Supplementary Figure S3). Indeed, mIgG and collagen I signals came up at 11 days post glycerol injection and the staining of eMHC showed a basal expression of this regeneration marker in saline-treated muscles. Finally, in glycerol plus ASC-treated animals the number of CNFs continued to increase over time to around 71.4 ± 15.8% (mean ± SEM, *n* = 3 muscles) at day 11 post injection, while it remained low in corresponding saline controls (0.6 ± 0.7%, mean ± SEM, *n* = 3 muscles). In summary, these results suggested that ASC application induced a late-phase inflammatory response and basal regenerative processes.

### ASC Transiently Mitigate Loss of Pre-synapses in the Glycerol Model

**FIGURE 4:**
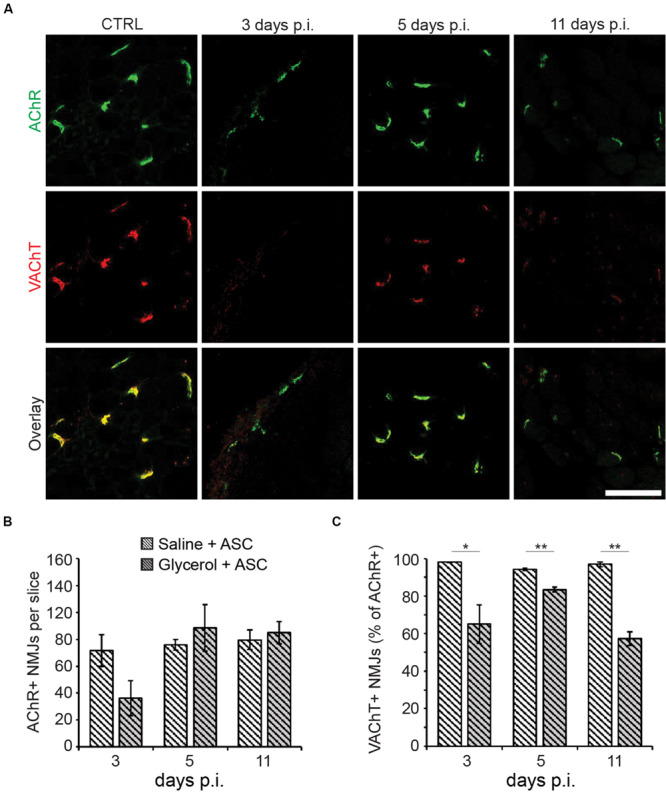
ASCs enhance early recovery of presynaptic VAChT enrichment, but this effect is transient. Simultaneous to tail-vein injection of ASCs, TA muscles were injected with 20 μl of either saline or glycerol and then harvested and snap frozen after 3, 5, or 11 days (days p.i.). Upon cryosectioning, muscle slices were stained with αBGT (for AChR detection) and antibodies against VAChT to label post- and presynaptic portions of NMJs, respectively. Sections were analyzed by confocal microscopy. **(A)** Representative optical sections of fluorescence signals as indicated, αBGT in green, VAChT in red, yellow in overlay images indicates colocalization of both signals. CTRL, saline-injected muscles at 3 days p.i., the other panels depict glycerol-injected muscles at 3, 5, and 11 days p.i., as indicated. Scalebar, 100 μm. **(B,C)** Quantitative analysis of αBGT+ postsynaptic sites per slice **(B)** and of VAChT+ NMJs (% of αBGT+ structures, **C**) as a function of days p.i. Shown is mean ± SEM (*n* = 3 muscles). *p ≤ 0.05, **p ≤ 0.01.

Finally, the effects of ASC treatment on NMJs were quantified. Therefore, muscle slices from animals treated with glycerol plus ASCs or saline plus ASCs were labeled for AChR and VAChT and then analyzed with confocal microscopy (Figure 4A). Counting of AChR+ sites did not reveal any significant differences between glycerol and saline injected muscles (Figure 4B) although at day 3 a trend toward lower numbers of postsynaptic sites in glycerol samples was visible. Quantitative analysis of AChR and VAChT double positive NMJs showed a good recovery at days 3 and 5 post injection. Indeed, while the fractions of double-positive NMJs in mice not treated with ASCs were low at these timepoints (day 3: 41.4 ± 17.3%; day 5: 48.1 ± 7.2%; mean ± SEM, *n* = 3 muscles), they reached 65.1% ± 10.3% and 83.4 ± 1.3% (mean ± SEM, *n* = 3 muscles) upon ASC injection. However, in ASC-treated mice, the numbers dropped at day 11 to a value of 57.3 ± 3.7% (mean ± SEM, *n* = 3 muscles) (Figure 4C), instead of a further recovery as observed in ASC-untreated animals (day 11: 65.8 ± 16.9%; mean ± SEM, *n* = 3 muscles). Thus, although ASC treatment showed some beneficial effect on NMJ recovery at early timepoints, this effect did not persist.

## Discussion

Stem cells have become a widely studied option to treat neuromuscular disorders. However, while their effects on the sarcomeric part of muscles have been investigated regularly, little is known on their regenerative capacity with respect to NMJs. In this study, a glycerol-based muscle damage model was employed to address three biological questions: (i) If a low dose of glycerol could trigger a degeneration – regeneration process, (ii) how this affected NMJs, and (iii) if and how a systemic treatment with ASCs could modulate muscle and synapse recovery.

### Low-Dose Application of Glycerol to Study Skeletal Muscle Degeneration-Regeneration

**FIGURE 5:**
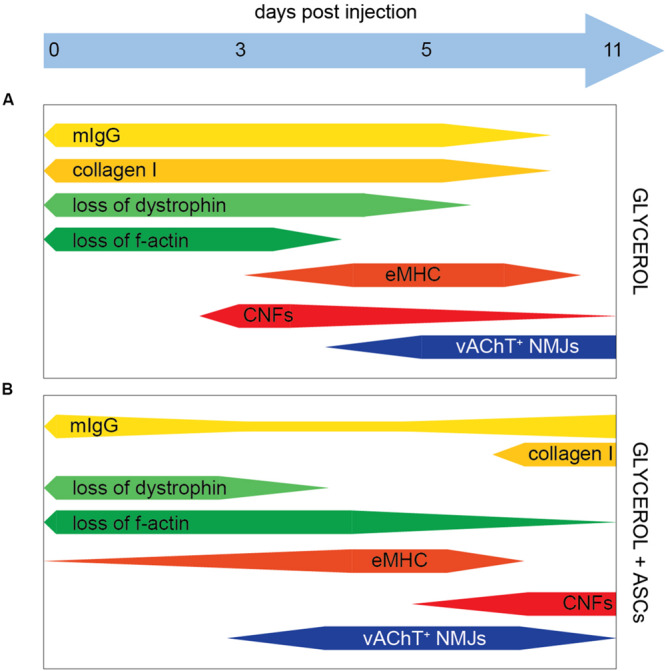
Schematic summary of glycerol and ASC effects. Bars indicate the presence of markers as indicated, the relative amount is shown by the bar width. **(A,B)** In the presence of Glycerol and Glycerol + ASC, respectively.

Most studies based on glycerol damage applied quantities of 50–100 μl of glycerol per mouse TA muscle (Lluís et al., 2001; Mahdy et al., 2015; Mahdy, 2018). This protocol was also used to induce massive rhabdomyolysis, in particular for the investigation of acute kidney disease (Korrapati et al., 2012; Huang et al., 2018; Reis et al., 2019). However, since we were more interested in the effects of ASCs on muscle and NMJ regeneration, a milder protocol was sought. One group reported that a dose of 25 μl of glycerol is able to cause a limited damage in mouse TA muscles including adipocyte infiltration, fibrosis, and necrosis (Arsic et al., 2004). In the present work, 20 μl per TA muscle were injected and this led to a consistent profile of local muscle degeneration and regeneration (Figure 5A). Regularly, necrosis was evident at 3 days post injection with loss of plasma membrane integrity, dystrophin, and f-actin/contractile apparatus in the affected area. Thereafter, the regeneration marker, eMHC, appeared between days 5 and 8 post injection, and at day 11, all parameters returned to normal except a persisting presence of CNFs. Thus, this paradigm appeared apt for investigating effects of ASC injection on NMJ regeneration.

### Effects of Glycerol on NMJ Degeneration-Regeneration

An analysis of post- and pre-synapses in the presence and absence of glycerol showed that the two synaptic compartments responded differently to the treatment: while a small but insignificant loss of post-synapses was detected over the entire experimental period, the pre-synapse marker VAChT was missing in around 60% of postsynaptic regions at 3 days post glycerol injection and then steadily recovered at the later timepoints (Figure 5A). A more in-depth analysis showed that the distribution of VAChT and AChR signal colocalization was similar between glycerol and saline injected muscles except for one trait: in the presence of glycerol, much more completely VAChT-negative synapses were found (Supplementary Figure S4), suggesting that the loss and reestablishment of presynaptic signals was not gradual but occurred in a rather digital manner. However, presynaptic regeneration in all synapses was not obtained within 11 days post injection. It is unclear, why pre- and postsynapse showed a differential susceptibility to glycerol. One possible hypothesis is that the presynaptic terminal as such was more prone to glycerol-induced damage than the postsynaptic part. Another possibility could be due to the focal glycerol-induced damage: it could be that at these sites it led to damage of nerve bundles upstream of undamaged muscle regions. If these damaged bundles were innervating a muscle region unaffected by glycerol, this could, by axon degeneration, affect the presynaptic elements also in apparently unaffected muscle regions. Interestingly, post-synapses were often found also in the most heavily damaged areas in muscle fibers that were infiltrated by mIgG and had lost their dystrophin and f-actin expression. Conversely, VAChT-positive NMJs were hardly detected in these areas. We have no formal proof that these same postsynaptic sites were later on reinnervated, but at least the rather constant amounts of postsynaptic sites obtained throughout the experiment argue against a massive phase of ectopic synapse formation. Depending on the type of damage model, different parts of a skeletal muscle can be affected. For example, treatments like mechanical, thermal, or ischemic injury induce a degeneration of cytoplasm and plasma membrane, but will leave the extracellular matrix largely intact (McMahan et al., 1980; Slater and Schiaffino, 2008; Anderson et al., 2017). Conversely, studies using glycerol showed that this kind of treatment leads to an extensive damage of the extracellular matrix (Hardy et al., 2016; Mahdy, 2018). Previously, it was described that an intact extracellular matrix is important for postsynaptic preservation and that it can efficiently guide motor axons to original synaptic sites (McMahan et al., 1980). Instead, the present study suggests that the loss of extracellular matrix is not sufficient to disperse postsynaptic assembly of AChR, but it might result in an impaired synapse recovery and pre-postsynaptic matching (Warren et al., 2007; Vilmont et al., 2016; Rogers and Nishimune, 2017). Nonetheless, a partial recovery of pre-postsynaptic matching was found here to start around day 5 post glycerol injection (Figure 5A). Compared to previous reports using either tourniquet (Tu et al., 2017) or nerve injury (Zainul et al., 2018), recovery was apparently faster here. In both previous studies, presynaptic recovery took weeks to complete. One might speculate that the nerve damage was only local in the case of glycerol injection, while it was global in the other models, which could potentially explain the different time courses observed. Thus, having achieved a robust series of degeneration and regeneration processes, we continued in investigating effects of ASC injection on NMJ regeneration.

### Effects of ASCs on De/Regeneration of Muscle and NMJs

Adipose-derived stromal cells were previously found to be interesting for clinical application due to their differentiation capacity, their beneficial effects on other cell types involved in tissue regeneration, and their immunomodulatory properties. In the present work, systemic ASC injection plus local glycerol application led to two major differences in the muscle degeneration – regeneration profiles as compared to glycerol injection alone: First, the reduced amounts of mIgG and collagen I as well as the early detection of eMHC and presynapse regeneration jointly indicated a slightly less severe damage at day 3 post glycerol injection. Second, however, the increasing amounts of mIgG, fibrosis, and CNFs at day 11 post glycerol suggested a secondary damage or differing regeneration phase. The latter effect of ASC application might be due to a mobilization of immune and stem cells into the muscle, causing a stronger regeneration phase. Initially, ASC may dampen the initial degeneration and inflammation by reducing the degree of necrosis and consequently the intensity of neutrophil-induced inflammation. In the following, they may impact the repair and regeneration phase for instance by an IL-6 dependent mechanism, as direct injection of mesenchymal stem/stromal cells was found to increase muscle regeneration via promoting the switch from M1 to M2 macrophages, hence, stimulating muscle stem cell activity (Arnold et al., 2007; Saclier et al., 2013; Mahdy, 2018). Finally, they may affect the remodeling of the regenerating muscle and the maturation of muscle cells, perhaps by a direct effect of ASCs migrating to affected muscles as reported previously (Vieira et al., 2012). Thus, ASCs could directly act on muscle recovery and enhance muscle regeneration (Rodriguez et al., 2005; Rybalko et al., 2017). By immunostaining of human nuclear marker Ku80 (Koike et al., 1999; Allard et al., 2014), we addressed settlement of systemically applied ASCs in muscles (Supplementary Figure S5). This did not reveal any clear sign of substantial integration of ASCs in glycerol-treated muscle tissue, rather suggesting that paracrine factors released by ASCs were involved in the observed modulation of degeneration – regeneration processes and immune responses. Recent findings on the effects of ASC secretome injection upon ischemic muscle damage favor this as a principal therapeutic mechanism (Mitchell et al., 2019; Figliolini et al., 2020). Here, ASC-derived exosomes promoted vascular growth and tissue regeneration. With respect to NMJs, two principal differences between mice treated with or without ASC were noted. First, upon application of saline, a rise in the number of postsynaptic sites per muscle slice was observed at day 11 p.i. in the absence (Figure 2B) but not in the presence of ASCs (Figure 4B). At present, the reason for this discrepancy is unclear. Likely, this was not due to general effects of ASC treatment on muscle integrity, since all other measured parameters, i.e. presence of mIgG, collagen I, dystrophin, actin, eMHC, fiber number, and CNFs, were unchanged. This suggests, that the observed difference was due to either, experimental variability or a specific effect of the ASCs on NMJ regeneration that needs further investigation. Second, upon injection of glycerol, ASC administration led to a fast recovery of post-synapse numbers within 5 days post injection and by trend also to a higher and faster presynapse recovery than in the absence of ASCs. The latter aspect, though, was reverted, likely due to the late-phase degenerative response, as indicated by enhanced levels of mIgG and collagen I on day 11 p.i. (Figure 5).

## Conclusion

In conclusion, although our data suggest a partially positive effect of ASCs on the recovery of NMJs in the early regenerative phase, a secondary degenerative or aberrant regenerative period reverted these benefits at a later stage. Due to the manifold effects of ASCs on various phases of de- and regeneration, which were likely involving muscle and neuronal cells, endothelial cells, but also all kind of immune cells, future studies need to further explore the mechanisms involved and work around late degenerative processes.

## Data Availability Statement

The raw data supporting the conclusions of this article will be made available by the authors, without undue reservation, to any qualified researcher.

## Ethics Statement

The animal study was reviewed and approved by the Regierungspräsidium Karlsruhe.

## Author Contributions

NG and RR contributed to conception and design of the study. MR, SH, and TS performed the experiments and statistical analysis. MR and RR wrote the first draft of the manuscript. KB and MH contributed to resources and materials. All authors contributed to manuscript revision and, read and approved the submitted version.

## Conflict of Interest

The authors declare that the research was conducted in the absence of any commercial or financial relationships that could be construed as a potential conflict of interest.
