# Supplementary material for: Effects of ASC Application on Endplate Regeneration Upon Glycerol-Induced Muscle Damage
Source: Front Mol Neurosci. 2020 Jun 23;13:107. doi: 10.3389/fnmol.2020.00107 (PMC7324987; doi:10.3389/fnmol.2020.00107)
Supplement: Supplementary file 1 [file Table_1.docx]

Supplementary Table 1

| Days post  injection | No ASCs | | ASCs | |
| --- | --- | --- | --- | --- |
|  | Glycerol | Saline | Glycerol | Saline |
| 3 | 46.25 ± 4.33;  185 (n=4) | 78.0 ± 20.6;  312 (n=4) | 36.3 ± 13.1;  101 (n=3) | 71.5 ± 11.79;  213 (n=3) |
| 5 | 67.3 ± 24.4;  202 (n=3) | 94.3 ± 15.7;  283 (n=3) | 88.5 ± 17.2;  254 (n=3) | 76.0 ± 3.9;  232 (n=3) |
| 8 | 59.3 ± 10.7;  178 (n=3) | 79.33 ± 17.42;  238 (n=3) | - | - |
| 11 | 44.3 ± 15.8;  133 (n=3) | 119.7 ± 19.2;  359 (n=3) | 85.0 ± 8.1;  255 (n=3) | 79.66 ± 7.17;  239 (n=3) |

Number of NMJs per experimental condition. Shown is mean ± SEM per muscle; number of NMJs found in total per condition (n indicates number of analyzed muscles – equals number of mice – per condition).
