# Supplementary material for: Effects of ASC Application on Endplate Regeneration Upon Glycerol-Induced Muscle Damage
Source: Front Mol Neurosci. 2020 Jun 23;13:107. doi: 10.3389/fnmol.2020.00107 (PMC7324987; doi:10.3389/fnmol.2020.00107)
Supplement: Supplementary file 2 [file Table_2.docx]

Supplementary Table 2

|  | No ASC | | With ASC | |
| --- | --- | --- | --- | --- |
| Days post  injection | Saline | Glycerol | Saline | Glycerol |
| 3 | 2010.5 ± 104.8 | 2136.5 ± 164.4 | 1852.4 ± 64.6 | 2028.0 ± 209.5 |
| 5 | 1885.6 ± 290.3 | 1969.0 ± 312.1 | 2234.0 ± 129.6 | 2075.5 ± 145.1 |
| 11 | 1934.6 ± 122.1 | 1963.1 ± 218.2 | 1836.1 ± 132.5 | 1651.3 ± 59.2 |

Cross-sectional area per fiber in µm^2^ per experimental condition. Shown is mean ± SEM (n=4 mice for No ASC 3 days post injection, n=3 mice for all other conditions). None of the values differed significantly from any other.
