# Supplementary material for: Effects of ASC Application on Endplate Regeneration Upon Glycerol-Induced Muscle Damage
Source: Front Mol Neurosci. 2020 Jun 23;13:107. doi: 10.3389/fnmol.2020.00107 (PMC7324987; doi:10.3389/fnmol.2020.00107)
Supplement: Supplementary file 3 [file Table_3.docx]

Supplementary Table 3

| Days post  injection | Saline | Glycerol |
| --- | --- | --- |
| 3 | 122.6 ± 38.6 | 203.5 ± 26.4 |
| 5 | 158.0 ± 33.7 | 180.3 ± 33.8 |
| 11 | 184.7 ± 20.3 | 158.2 ± 42.4 |

Areas of NMJs in µm^2^ per experimental condition. Shown is mean ± SEM (n=3). None of the values differed significantly from any other.
