# Supplementary material for: Effects of ASC Application on Endplate Regeneration Upon Glycerol-Induced Muscle Damage
Source: Front Mol Neurosci. 2020 Jun 23;13:107. doi: 10.3389/fnmol.2020.00107 (PMC7324987; doi:10.3389/fnmol.2020.00107)
Supplement: Supplementary file 4 [file Image_1.pdf]

Supplementary Figure 1

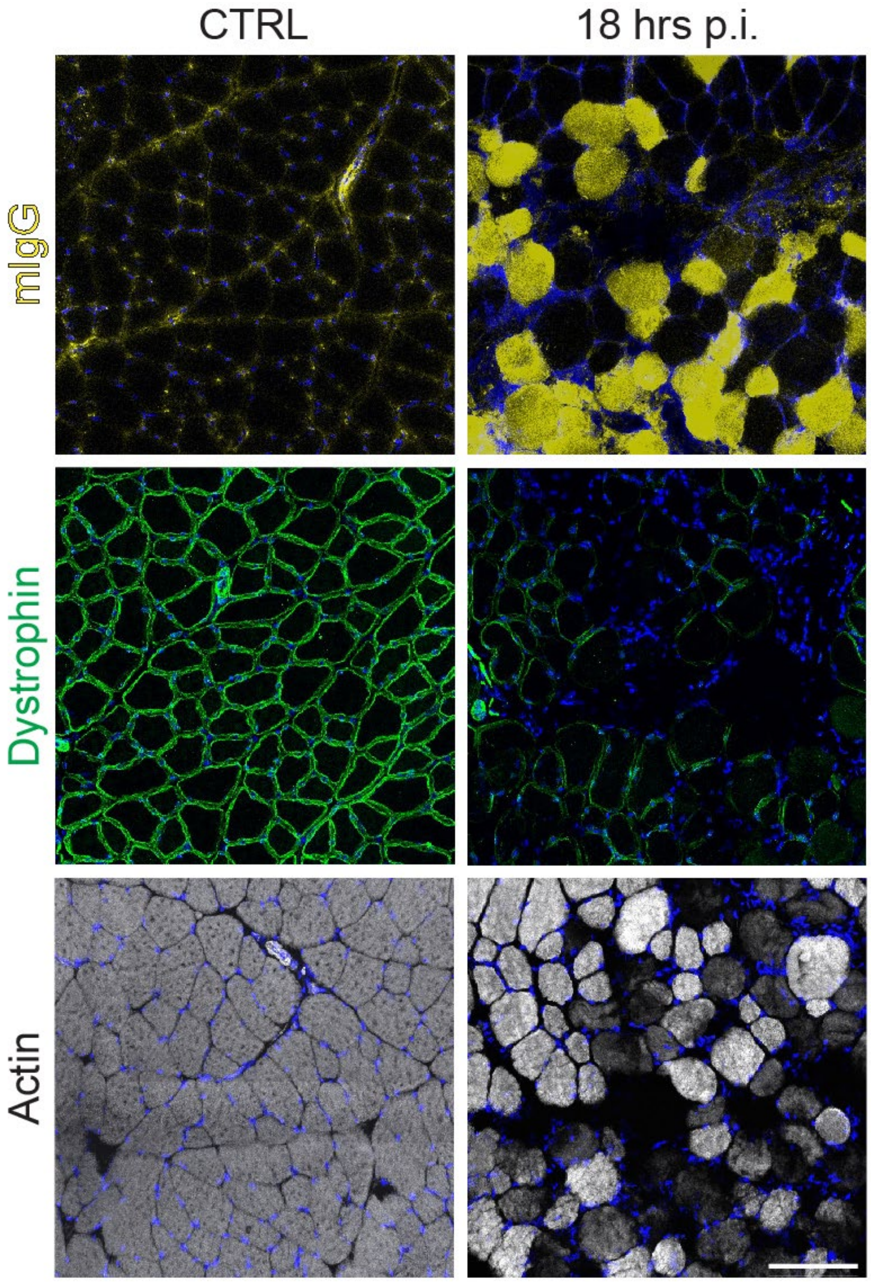

Muscle damage is visible few hours after glycerol injection. TA muscles were injected with 20 µl of either saline or glycerol and then harvested and snap frozen after 18 hours. Upon cryosectioning, muscle slices were stained with DAPI and either antibodies against mouse IgG (mIgG), dystrophin, or with phalloidin-TRITC to label actin. Sections were analyzed by confocal microscopy. Shown are representative optical sections of fluorescence signals as indicated, nuclear DAPI staining always shown in blue, mIgG in yellow, dystrophin in green, actin in grey. CTRL, saline-injected muscles at 18 hours p.i. Scalebar, 100 µm.
