# Supplementary material for: Effects of ASC Application on Endplate Regeneration Upon Glycerol-Induced Muscle Damage
Source: Front Mol Neurosci. 2020 Jun 23;13:107. doi: 10.3389/fnmol.2020.00107 (PMC7324987; doi:10.3389/fnmol.2020.00107)
Supplement: Supplementary file 5 [file Image_2.pdf]

Supplementary Figure 2

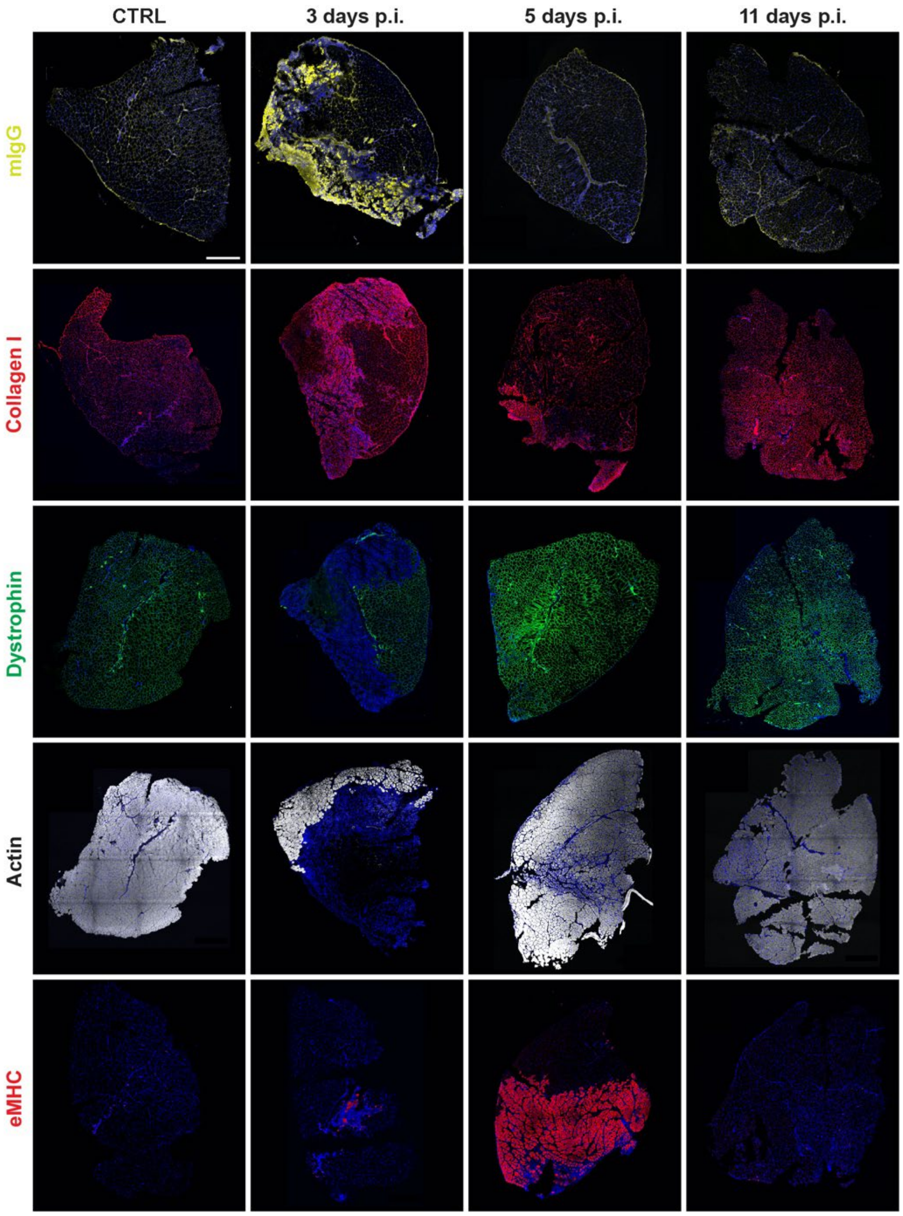

Glycerol-induced muscle damage leads to early necrosis, loss of dystrophin and actin, transient eMHC appearance, and prolonged presence of center-nucleated fibers. TA muscles were injected with 20 µl of either saline or glycerol and then harvested and snap frozen after three, five, or eleven days (days p.i.). Upon cryo-sectioning, muscle slices were stained with DAPI and either antibodies against mouse IgG (mIgG), collagen I, dystrophin, or embryonic myosin heavy chain (eMHC), or with phalloidin-TRITC to label actin. Sections were analyzed by confocal microscopy. Fluorescence signals in whole crysoections as indicated, nuclear DAPI staining always shown in blue, mIgG in yellow, collagen I and eMHC in red, dystrophin in green, actin in grey. CTRL, saline-injected muscles at three days p.i., the other panels depict glycerol-injected muscles at three, five, and eleven days p.i., as indicated. Scalebar, 500 µm.
