# Supplementary material for: Effects of ASC Application on Endplate Regeneration Upon Glycerol-Induced Muscle Damage
Source: Front Mol Neurosci. 2020 Jun 23;13:107. doi: 10.3389/fnmol.2020.00107 (PMC7324987; doi:10.3389/fnmol.2020.00107)
Supplement: Supplementary file 6 [file Image_3.pdf]

Supplementary Figure 3

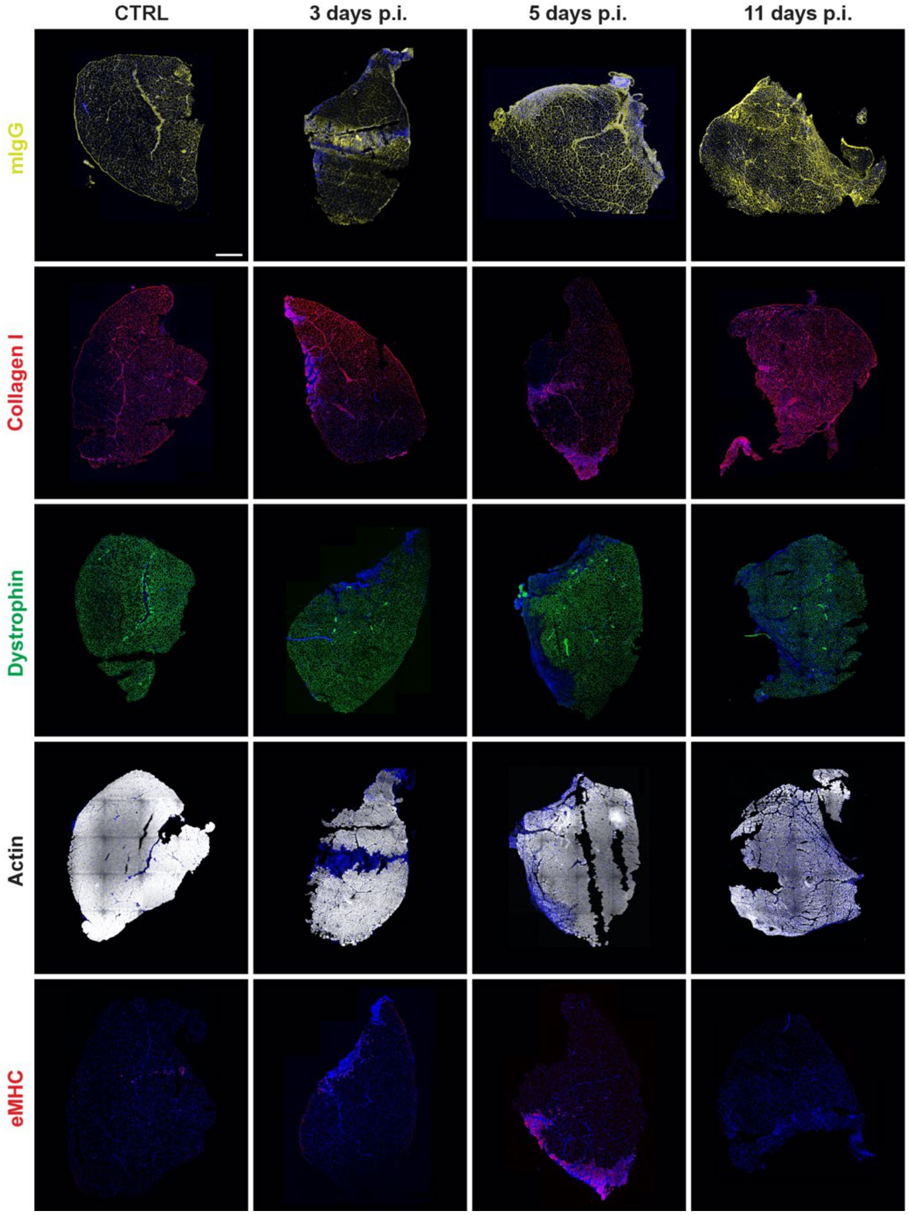

Systemic injection of ASCs induces a late phase of IgG infiltration, enhanced basal eMHC expression, and increase in center-nucleated fibers. Simultaneous to tail-vein injection of ASCs, TA muscles were injected with 20 µl of either saline or glycerol and then harvested and snap frozen after three, five, or eleven days (days p.i.). Upon cryosectioning, muscle slices were stained with DAPI and either antibodies against mouse IgG (mIgG), collagen I, dystrophin, or embryonic myosin heavy chain (eMHC), or with phalloidin-TRITC to label actin. Sections were analyzed by confocal microscopy. Fluorescence signals in whole cryosections as indicated, nuclear DAPI staining always shown in blue, mIgG in yellow, collagen I and eMHC in red, dystrophin in green, actin in grey. CTRL, saline-injected muscles at three days p.i., the other panels depict glycerol-injected muscles at three, five, and eleven days p.i., as indicated. Scalebar, 500 µm.
