# Supplementary material for: Effects of ASC Application on Endplate Regeneration Upon Glycerol-Induced Muscle Damage
Source: Front Mol Neurosci. 2020 Jun 23;13:107. doi: 10.3389/fnmol.2020.00107 (PMC7324987; doi:10.3389/fnmol.2020.00107)
Supplement: Supplementary file 7 [file Image_4.pdf]

Supplementary Figure 4

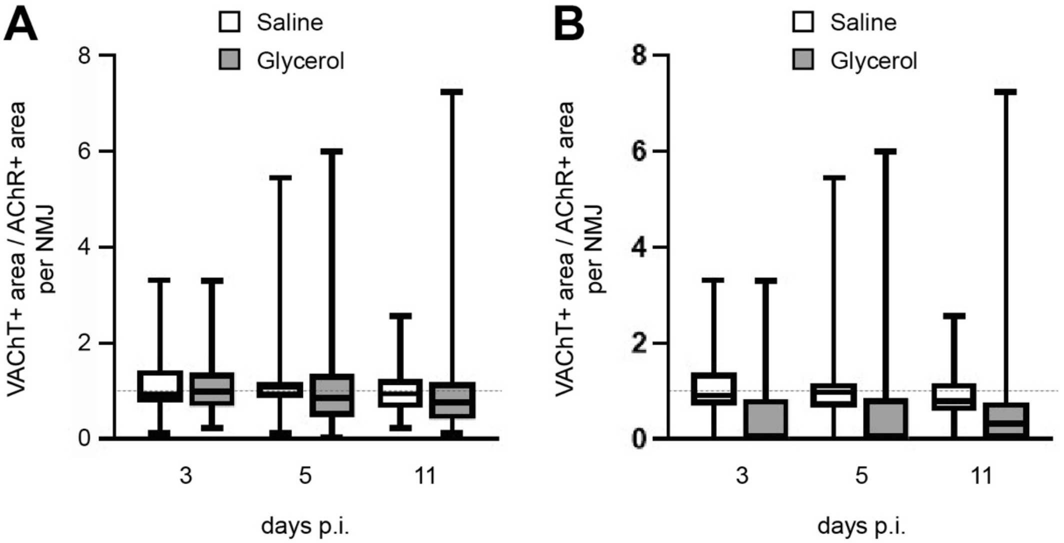

Loss and recovery of innervation in glycerol-injected muscles occurs in an all-or-none fashion. TA muscles were injected with 20 µl of either saline or glycerol and then harvested and snap frozen after three, five, or eleven days (days p.i.). Upon cryosectioning, muscle slices were stained with αBGT (for AChR detection) and antibodies against VAChT to label post- and presynaptic portions of NMJs, respectively. Sections were analyzed by confocal microscopy. Graphs show Box-and-Whisker plots of the VAChT+ presynaptic area normalized to the corresponding αBGT+ postsynaptic area. A value of 1 (dashed line) indicates perfect match, higher and lower values show either larger or smaller presynaptic vs. postsynaptic area, respectively. (A) Values leaving out or (B) including completely VAChT- NMJs. Number of analysed muscles (number of analysed NMJs / number of completely VAChT- NMJs): Saline 3 d.p.i.: 3(55/2), 5 d.p.i.: 4(78/5), 11 d.p.i.: 3(77/2); Glycerol 3 d.p.i.: 4(121/71), 5 d.p.i.: 4(91/47), 11 d.p.i.: 3(97/33).
