# Supplementary material for: Effects of ASC Application on Endplate Regeneration Upon Glycerol-Induced Muscle Damage
Source: Front Mol Neurosci. 2020 Jun 23;13:107. doi: 10.3389/fnmol.2020.00107 (PMC7324987; doi:10.3389/fnmol.2020.00107)
Supplement: Supplementary file 8 [file Image_5.pdf]

Supplementary Figure 5

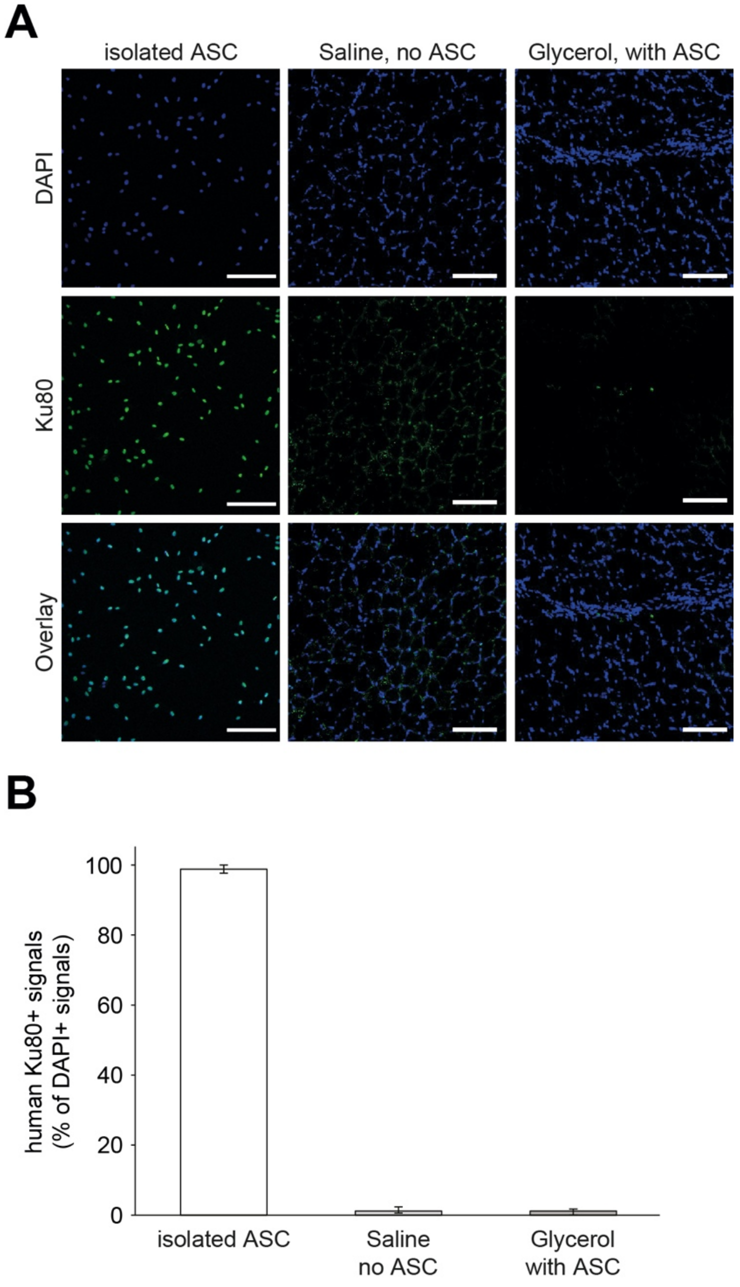

Integration of systemically injected ASC into muscle is not detectable by immunofluorescence. Isolated human ASC and mouse TA muscle sections taken five days post injection of either saline or glycerol (indicated) were stained with DAPI and an anti-human Ku80 antibody against DNA and human nuclei, respectively. (A) Representative confocal micrographs showing fluorescence signals of DAPI and human Ku80 in blue and green, respectively. Scale bars, 100 µm. (B) Quantitative analysis of the percentage of human Ku80+ signals found in DAPI+ nuclei.
